# The Relationship Between Diet and the Neuropathological Hallmarks of Alzheimer’s Disease in Cognitively Normal Adults: A Systematic Narrative Review

**DOI:** 10.3390/healthcare13202628

**Published:** 2025-10-20

**Authors:** Amanda A. Harb, Kelly A. Brock-Spano, Jill R. Silverman, Jack R. Thomas, Ashley S. Pothen

**Affiliations:** Department of Nutrition Science and Wellness, Farmingdale State College, 2350 Broadhollow Rd., Farmingdale, NY 11735, USA

**Keywords:** Alzheimer’s disease, biomarkers, diet, prevention

## Abstract

**Background/Objectives**: Alzheimer’s disease (AD) remains a leading cause of mortality with millions suffering worldwide. The number of annual cases is sharply increasing primarily due to growing older adult populations. To date, there is neither an effective prevention nor cure for AD. Recently, AD was specified using biomarkers, facilitating research into primary and secondary prevention strategies, including dietary interventions. This systematic narrative review maps the literature on dietary prevention of AD by synthesizing the evidence on diet and AD biomarkers in cognitively normal adults. Additionally, it explores limitations in the current evidence base and identifies areas for future research. **Methods**: Search terms and inclusion/exclusion criteria were set, and PubMed and EBSCOhost were searched for articles up through September 2025. Out of 331 results, 14 articles passed the inclusion/exclusion criteria and were included in this review. **Results**: Most studies were cross-sectional (n = 8), followed by cohort (n = 4), with one study including both cross-sectional and longitudinal analyses (n = 1). Only one intervention study was published. Various dietary exposures were tested, with most studies (n = 5) supporting a protective relationship between the Mediterranean diet and prevention of the neuropathological hallmarks of AD. However, the evidence base varies in methodology. Future research would benefit from greater consensus in methodology and should prioritize prospective cohort and randomized trial designs. **Conclusions**: Evidence from this review suggests existence of a potential role for dietary interventions, especially the Mediterranean diet, in AD prevention. However, further research is needed to address existing gaps. (248 words)

## 1. Introduction

Alzheimer’s disease (AD) is a neurodegenerative disorder that causes dementia, which is a state of cognitive decline across several functions, beginning with short-term memory but progressing to other cognitive functions including reasoning, judgement, language, and visuospatial and motor functions [1,2]. According to a 2025 World Health Organization fact sheet, 60–70% of dementia cases are at least partly caused by AD [3]. There are two types of AD: familial AD and sporadic AD. Familial AD is less prevalent (5–15% of cases) and has known genetic causes. Sporadic AD is more prevalent (85–95% of cases); the causes are unknown, but several risk factors have been identified, including genetic risk factors such as the presence of two apolipoprotein E (APOE) epsilon 4 (ε4) alleles [1]. AD is considered a disease of old age as the incidence and prevalence of AD increase markedly with increased age [2]. There are also sex differences: the prevalence of AD is higher among women compared to men [1]. The prevalence of AD is projected to increase, but there remains no cure [2]. The current prevalence of AD, the projected increased prevalence, and the lack of a cure has led to an interest in primary and secondary prevention strategies [4], especially dietary strategies.

There are many observational studies on diet and AD prevention, but few clinical trials focused on cognitively normal adults. Systematic reviews and meta-analyses of prospective cohort studies report a relationship between diet and AD incidence, revealing diet as a modifiable risk factor of AD. These studies have identified protective dietary factors against AD, including fish consumption [5,6,7], dietary (not supplemental) vitamin C intake [8], and antioxidant intake [9]. In terms of dietary patterns, a 2025 meta-analysis of cohort studies found that the Mediterranean diet, the Dietary Approaches to Stop Hypertension (DASH) diet, and the Mediterranean-DASH Intervention for Neurodegenerative Delay (MIND) diet protect against dementia [10]. However, evidence from trials on cognitively normal adults is more limited. While there is some evidence that diet may prevent AD when part of a multidomain lifestyle intervention [11,12], the effect of diet alone is less clear, with one 2023 trial study showing no significant effect of the MIND diet on cognitive and brain imaging outcomes [13]. The paucity of trials is understandable given the long latency period of AD, making trials on prevention strategies impractical to perform. The biomarker-based definition of AD overcomes this challenge to AD prevention trials, making them more feasible.

An understanding of the pathophysiology of AD is necessary to identify predictive or pre-symptomatic biomarkers that can serve as targets in research on primary and secondary prevention. There are two neuropathological features that distinguish AD from other causes of dementia: beta-amyloid plaques and neurofibrillary tangles [2,14,15]. These features are referred to as the “hallmarks” of AD [1,16]. They are microscopic, but there are also macroscopic features of AD.

One of the macroscopic features, or “grossly visible lesions,” is atrophy in the cerebral cortex, especially in the “frontotemporal association cortex,” but largely not in the primary motor, sensory, and visual areas [14,17]. The frontotemporal association cortex refers to the cortical association areas in the frontal and temporal lobes. The general function of association areas is integration of sensory inputs and motor outputs [18]. As stated by Purves et al. (2001) [18], “[t]he diverse functions of the association cortices are loosely referred to as ‘cognition,’ which literally means the process by which we come to know the world,” and dementia is indeed a decline in this cognitive function [1,2]. Other macroscopic features of AD are increased sulcal width [19] and enlargement of the ventricles in the brain [14].

However, macroscopic features of AD are non-specific. They may be found in elderly people with normal cognitive function or in people with dementia caused by diseases other than AD. The presence of AD is suggested if both of the following observations are made: (1) there is “significant” atrophy in the hippocampus, and (2) the inferior horns of the lateral ventricles are enlarged [14]. Nonetheless, even with the presence of these features, AD cannot be diagnosed until a microscopic examination is performed.

Two microscopic features are required for the diagnosis of AD. One of these microscopic features is the presence of beta-amyloid plaques [14]. These plaques are found outside the neurons [15]. According to Serrano-Pozo et al. (2011) [17], the plaques spread in three stages: stage one is in the neocortex; stage two is in the allocortex and limbic structures; and stage three is in various subcortical structures, including structures in the basal forebrain, brainstem, pons, and cerebellum. The other microscopic feature required for AD diagnosis is the presence of neurofibrillary tangles [14]. These tangles are found inside the neurons [15]. According to Serrano-Pozo et al. (2011) [17], the tangles also spread in three stages: stage one is in the transentorhinal and entorhinal cortices, which are in the medial temporal lobe; stage two is in the limbic structures; and stage three is in the neocortex, especially in the associative areas.

While the hallmarks of AD are known, the neuropathogenesis of AD remains unknown. However, several hypotheses have been proposed. The most tested hypotheses are the cholinergic hypothesis, the amyloid hypothesis, and the mitochondrial cascade hypothesis [20].

The cholinergic hypothesis proposes that AD pathogenesis begins with the loss of cholinergic neurons in the nucleus basalis of Meynert, which is in the basal forebrain. These cholinergic neurons project to the limbic system and neocortex, where they are a chief source of the neurotransmitter acetylcholine (ACh) [21,22]. In a non-diseased state, this neurotransmitter is synthesized in the pre-synaptic cholinergic neuron from acetyl CoA and then released from the pre-synaptic neuron into the synaptic cleft when the pre-synaptic neuron is depolarized in an action potential. After ACh binds to either a nicotinic or muscarinic receptor on the post-synaptic neuron, there is a breakdown of the ACh remaining in the synaptic cleft, catalyzed by acetylcholinesterase (AChE) [22]. However, in AD, the pre-synaptic cholinergic neurons are damaged, including their axons to the cerebral cortex. The loss of these neurons is believed to lead to a loss of the nicotinic receptors in the neocortex and limbic system. The resulting lost cholinergic innervation is hypothesized to be responsible for the dementia caused by AD [21]. Notably, AChE inhibitors are one of the two classes of drugs used to manage the symptoms of AD. They work by inhibiting the breakdown of ACh in the synaptic cleft by AChE [20].

The amyloid hypothesis proposes that AD pathogenesis begins with the formation of amyloid-beta and plaques from amyloid precursor protein (APP). This protein is embedded in the cell membrane of neurons, and a part of APP is outside the cell membrane. In a non-diseased state, APP is cleaved by two enzymes: alpha-secretase and gamma-secretase. This leads to the formation of two soluble proteins: APPsα from alpha-secretase, and P3 from gamma-secretase. These proteins are found outside the neuron, while the remainder of APP stays in the cell membrane. However, in AD, APP is cleaved by beta-secretase and gamma-secretase. This leads to the formation of two different proteins: soluble APPsβ from beta-secretase, and insoluble amyloid-beta from gamma-secretase. Amyloid-beta proteins tend to combine outside the neuron to form beta-amyloid plaques. There are two types of amyloid-beta: Aβ40, which is less likely to form plaques, and Aβ42, which is more likely to form plaques. The amyloid-beta and resulting plaques cause several problems: interference with synaptic signaling, neuronal apoptosis, and activation of kinases that go on to phosphorylate tau proteins in the neurons. When they are not hyperphosphorylated, tau proteins are found inside the microtubules, where they provide support to the microtubules. However, when hyperphosphorylated, tau proteins tend to combine in the cytoplasm to form neurofibrillary tangles. The hyperphosphorylation of tau proteins in the microtubules and resulting insoluble tangles also cause neuronal apoptosis. Overall, the loss of synaptic signaling and neurons is hypothesized to be responsible for the dementia caused by AD [15,20]. However, it is important to note that some have argued that the plaques and tangles are not causes but products of neurodegeneration [23], though a 2023 study provides support for the amyloid hypothesis [24].

The mitochondrial cascade hypothesis proposes that AD pathogenesis begins with mitochondrial dysfunction [25]. When mitochondria are healthy, they provide energy to the neurons by oxidative phosphorylation, a pathway that takes place on the electron transport chain of the mitochondria. Mitochondria also protect the neurons from reactive oxygen species (ROS), which are byproducts of oxidative phosphorylation [26]. With age, oxidative phosphorylation decreases and ROS production increases. Our genes and environmental factors determine the rate at which this decline in mitochondrial function occurs [27]. Eventually, the decline surpasses a threshold, causing amyloid-beta formation from APP in response to ROS overproduction, neuronal apoptosis due to ROS overproduction and low oxidative phosphorylation, and tau hyperphosphorylation and neurofibrillary tangle formation in response to neurons trying to resume growth and division. The plaques, tangles, and resultant loss of neurons and synapses align with the microscopic features of AD, and these features are hypothesized to be responsible for the dementia caused by AD [25].

Whether a cause or product of neurodegeneration, plaques and tangles are the hallmarks of AD, and their biomarkers can serve as targets for prevention studies. In 2018, the NIA-AA released a revised research framework that defined AD using biomarkers. In the clinical setting, AD is diagnosed based on signs and symptoms as “probable or possible AD,” not definite AD. This is because a diagnosis of AD requires a microscopic examination of brain tissue that can only be performed after the patient passes. For the research setting, the 2018 NIA-AA framework argues for a definition of AD based on biomarkers only, not signs or symptoms. Among other things, this “biological definition” considers that (a) the hallmarks of AD are plaques and tangles, (b) AD is a progressive disease and exists on a continuum with a pre-symptomatic phase, (c) AD pathogenesis is still unknown, especially with respect to whether plaques and tangles are causes of neurodegeneration, and (d) AD is not a type of dementia but a cause of dementia. The authors of the framework argue that many diseases are defined by biomarkers, including hypertension and diabetes, and this type of definition has helped researchers identify interventions that can prevent disease progression, especially from a pre-symptomatic phase to a symptomatic phase [28].

The 2018 NIA-AA framework recognizes three groups of biomarker evidence (Table 1). The framework recommends measuring all biomarker outcomes as continuous variables. These biomarkers may be measured in cerebrospinal fluid (CSF) in the brain or by images of the brain generated using positron emission tomography (PET), fluoro-deoxyglucose (FDG) PET, and magnetic resonance imaging (MRI).

This grouping of biomarkers is referred to as the “AT(N) classification system”: Group A biomarkers provide evidence of beta-amyloid plaques; group T biomarkers provide evidence of hyperphosphorylated tau; and group (N) biomarkers provide evidence of neurodegeneration. Group (N) is in parenthesis to denote that these biomarkers are not hallmarks of AD and may be evidence of other causes of neurodegeneration. Biomarker evidence from both groups A and T are required to prove that an individual has AD, whether pre-symptomatic or symptomatic AD [28].

In 2024, an AA workgroup published a proposed revision of these criteria. This revision included the addition of three groups to the AT(N) framework: Group (I) biomarkers provide evidence of inflammation; group (V) biomarkers provide evidence of vascular brain injury; and group (S) biomarkers provide evidence of alpha-synucleinopathy. As indicated by the parentheses, none of these groups provide evidence of the hallmarks of AD. Furthermore, groups (V) and (S) indicate non-AD co-pathologies, like Parkinson’s disease. In addition to the new groups, the 2024 revision also included the addition of plasma biomarkers for groups T, (N), and (I); the removal of low Aβ42 alone from group A and high t-tau from group (N); the specification of phosphorylated tau isoforms; and the division of group T biomarkers into two groups, with T_1_ indicating early, soluble tau and T_2_ indicating later, tau aggregates [29]. Table 2 summarizes the 2024 framework.

Using the NIA-AA framework as a guide, the aim of this systematic narrative review is to map the literature to answer the following PICO question: in cognitively normal adults, what do we know about the relationship between diet and the development of the neuropathological hallmarks of AD? Cognitively normal adults were selected as the population because there is an interest in primary and secondary prevention of AD, which would target cognitively normal adults, whether not on the AD continuum or on the AD continuum but in a pre-symptomatic phase. Importantly, the recently developed NIA-AA framework allows us to focus on this population because it defined AD using biomarkers, allowing investigation into prevention of AD progression among cognitively normal adults.

There have been other reviews on diet and AD biomarkers. In 2019, a systematic review and meta-analysis investigating diet and its effects on AD biomarkers found a small, significant effect; however, this review did not focus on cognitively normal adults [30]. A 2022 review focused on dietary patterns only, not individual nutrients, and their relationship with MRI biomarkers only [31], which are not biomarkers of AD hallmarks. A 2023 systematic review focused on the Mediterranean diet and neuroimaging biomarkers but did not include biomarkers from groups A and T [32]. To the best of our knowledge, our review is the first review on diet and AD prevention that is both focused on cognitively normal adults and the hallmarks of AD as defined using biomarkers in the 2018 NIA-AA framework and the 2024 revised framework. By identifying the trends and research gaps in the current evidence base, this systematic narrative review aims to advance the research on primary and secondary prevention of AD.

## 2. Materials and Methods

### 2.1. Design

A systematic narrative review design was selected due to the breadth of the prevention strategy being examined. This review does not focus on a single dietary prevention strategy but includes any diet-related intervention or exposure. This flexibility introduces heterogeneity that is better addressed by a systematic narrative review.

### 2.2. Search Strategy

Two platforms were used to search for literature up through September 2025: PubMed and EBSCOhost. In consultation with a scholarly communication librarian, the following databases were selected: MEDLINE, Biological and Agricultural Index Plus (H.W. Wilson), CINAHL Plus, General Science (H.W. Wilson), and Health Source- Nursing and Academic Edition. The PubMed search was conducted using the filters for human subjects only and English only along with the following keywords: diet AND Alzheimer Disease AND biomarker. This search returned 282 results. The EBSCOhost search was conducted using the filters for Peer Reviewed only and English only along with the following keywords: (diet OR diets) AND (“Alzheimer disease” OR Alzheimer) AND (biomarker OR biomarkers) AND (MH “Humans” OR “human subjects” OR humans). This search returned 49 results.

### 2.3. Inclusion/Exclusion Criteria

The inclusion criteria were as follows: (1) the article must be a primary article, (2) the sample must consist of cognitively normal adults, (3) the intervention or exposure is diet-related, and (4) the primary outcomes include at least one of the biomarkers in either the 2018 NIA-AA framework or the 2024 revised framework. Studies were excluded if (1) cognitively normal adults were only used as the control group, (2) the outcomes included only biomarkers from the (N)/(I)/(V)/(S) groups, and (3) the study was not published in English. Notably, samples of cognitively normal adults may include adults not on the AD continuum or adults on the AD continuum but in the pre-symptomatic phase.

### 2.4. Study Selection and Data Extraction and Synthesis

**Figure 1:**
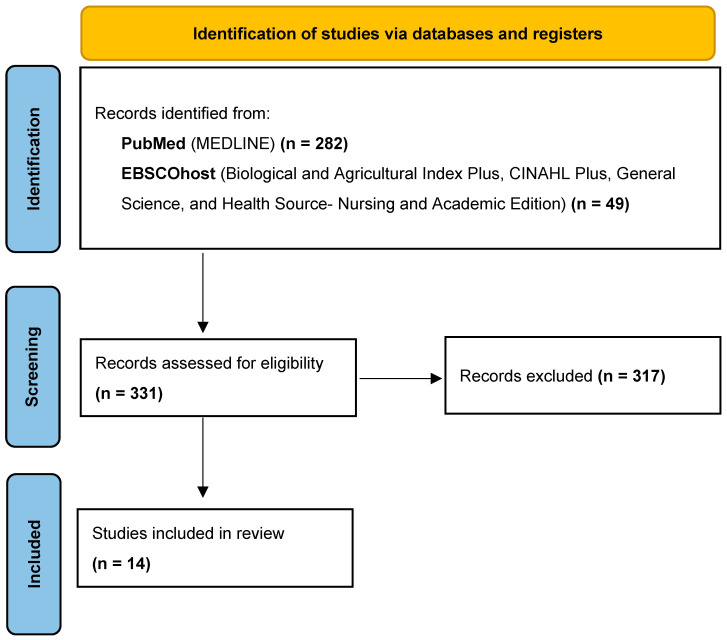
Modified “PRISMA 2020 flow diagram for new systematic reviews which included searches of databases and registers only” by the PRISMA Executive, licensed under CC BY 4.0 [33].

The first author reviewed all titles and abstracts. If the title or abstract did not clearly include an exclusion criterion, the full text was assessed. Results were shared with the fifth author, who confirmed that all studies that passed the inclusion/exclusion criteria were selected. Overall, 14 studies passed the inclusion/exclusion criteria (Figure 1). Data extracted from these studies included author, year, study design, sample characteristics (country, size, mean age, percent female, percent APOE ε4 +), the exposure/intervention variable along with the instrument used to measure it, the biomarker primary outcomes, covariates, and limitations. Studies were grouped by design for a narrative synthesis of the evidence to be presented in the results section.

## 3. Results

### 3.1. Cross-Sectional

Most of the studies had a cross-sectional study design (n = 8), with one study having both cross-sectional and longitudinal analyses (n = 1). All cross-sectional analyses are presented in this sub-section in chronological order by year of publication and in alphabetical order by last name of the first author. A summary of the studies is presented in Table 3.

In 2014, Mosconi et al. [34] published a study on the relationship between the intake of 10 nutrients and two imaging biomarkers: (1) amyloid load, measured using PET, and (2) glucose metabolism in neurons, measured using FDG-PET. Nutrient intake was measured using a semi-quantitative food frequency questionnaire (SQ-FFQ). The 10 nutrients were (1) saturated fatty acids (SFAs), (2) monounsaturated fatty acids (MUFAs), (3) omega-3 fatty acids (including eicosapentaenoic acid (EPA)), (4) omega-6 fatty acids, (5) beta-carotene, (6) vitamin B12, (7) vitamin C, (8) vitamin D, (9) vitamin E, and (10) folate. The regions of interest (ROIs) were the inferior parietal lobe, lateral temporal lobe, medial frontal gyrus, posterior cingulate cortex, and prefrontal cortex. The sample consisted of cognitively normal adults in New York City (n = 49, mean age = 54 years, 69% female, 39% APOE ε4 positive). Using linear regression (adjusted for age and energy intake), the researchers found negative associations between the following nutrients and average cortical amyloid load: vitamin B12 (β = −0.32, *p* ≤ 0.05) and EPA (β = −0.33, *p* ≤ 0.05). They also found positive associations between the following nutrients and glucose metabolism: beta-carotene (β = 0.31, *p* ≤ 0.05) and folate (β = 0.35, *p* ≤ 0.01). These main findings suggest that a diet high in vitamin B12 and EPA may be protective against amyloid plaques, while a diet high in beta-carotene and folate may be protective against neurodegeneration [34].

In 2015, Berti et al. [35] published a study on the relationship between five nutrient patterns and three imaging biomarkers: (1) amyloid load, measured using PET, (2) glucose metabolism, measured using FDG-PET, and (3) gray matter volume, measured using MRI. Nutrient intake was measured using a SQ-FFQ. The five nutrient patterns were (1) high vitamin B and minerals; (2) high MUFAs, polyunsaturated fatty acids (PUFAs), and vitamin E, (3) high vitamin A, vitamin C, carotenoids, and dietary fiber (DF); (4) high vitamin B12, vitamin D, and zinc; and (5) high SFA, trans-fat, cholesterol, and sodium. The ROIs were the posterior cingulate cortex, inferior and superior parietal lobules, lateral and medial temporal cortex, medial and prefrontal cortex, and striatum. The sample consisted of cognitively normal adults in New York City (n = 52, mean age = 54 years, 71% female, 47% APOE ε4 positive). Notably, these adults were sampled from the same cohort sampled by Mosconi et al. (2014) [34]. Using multiple regressions (adjusted for sex, education, APOE ε4 status, ethnicity, body mass index (BMI), alcohol intake, and family history), the researchers found negative associations between a nutrient pattern higher in vitamin B12, vitamin D, and zinc and amyloid load in several ROIs (*p* < 0.001). They also found positive associations between three nutrient patterns and glucose metabolism in the neurons in several ROIs: high MUFAs, PUFAs, and vitamin E (*p* < 0.001); high vitamin A, C, carotenoids, and DF (*p* < 0.001); and high vitamin B12, D, and zinc (*p* < 0.001). There were positive associations between two nutrient patterns and gray matter volume: high MUFAs, PUFAs, and vitamin E (*p* < 0.001); and high vitamin B12, vitamin D, and zinc (*p* < 0.001). The nutrient pattern high in fats was negatively associated with glucose metabolism (*p* < 0.001) and gray matter volume (*p* < 0.001). These main findings suggest that several nutrient combinations may be protective against amyloid plaques and neurodegeneration, while a nutrient pattern high in fat may increase risk of neurodegeneration [35].

In 2018, Fernando et al. [36] published a study on the relationship between protein and DF intake and imaging and plasma biomarkers: amyloid load, measured using PET, and Aβ42, Aβ40, and Aβ42/40 in plasma. Protein and DF intakes were measured using a SQ-FFQ. The ROIs were the frontal, superior parietal, lateral temporal, and lateral occipital lobes, and the anterior and posterior cingulate cortex. The outcome was measured as a dichotomous variable: high or low amyloid load [36]. Notably, the 2018 NIA-AA framework recommends measurement of biomarker outcomes as continuous variables [28]. The sample consisted of cognitively normal adults above age 60 in Australia (n = 541, mean age = 70 years, 59% female, 26% APOE ε4 positive). Using logistic regression (adjusted for age, sex, education, APOE ε4 status, born in Australia vs. another country, BMI, and energy intake), the researchers found that adults in the lowest tertile for protein intake (mean = 54 g/day) had 12.6 greater odds of high amyloid load compared to adults in the highest tertile (mean = 118 g/day) (OR = 12.594, 95% CI = 1.705, 93.018, *p* = 0.008). There was no significant association between DF intake and amyloid load and no significant associations between any of the exposures and plasma Aβ42, Aβ40, and Aβ42/40 [36].

In 2018, Vassilaki et al. [37] published a study on the relationship between adherence to the Mediterranean diet and amyloid load, measured using PET. Adherence to the Mediterranean diet was determined using a SQ-FFQ. The ROIs were the parietal and temporal lobes, prefrontal and orbitofrontal cortices, and posterior cingulate cortex and precuneus. The sample consisted of cognitively normal adults above age 70 in Minnesota (n = 278, mean age = 78 years, 44% female, 27% APOE ε4 positive). Using linear regression (adjusted for age, sex, education, APOE ε4 status, time from FFQ to PET, and energy intake), the researchers found a negative association between higher adherence to the Mediterranean diet and amyloid load (β = −0.035, 95% CI = −0.063, −0.008, *p* = 0.012). This finding suggests that the Mediterranean diet may be protective against amyloid plaques [37].

In 2020, Ma et al. [38] published a study on the relationship between frequency of green tea consumption and several CSF biomarkers: Aβ42, p-tau, t-tau, and ratios of CSF biomarkers. Green tea consumption was measured using one question on weekly consumption. The sample consisted of cognitively normal adults between ages 40–60 in China (n = 722, mean age = 62 years, 40% female, 15% APOE ε4 positive). Using multiple linear regressions (adjusted for age, sex, education, APOE ε4 status, and cognitive scores), the researchers found a negative association between green tea consumption frequency and t-tau (β = −0.00428, *p* = 0.041), but they found no associations for all the other biomarkers, including t-tau/Aβ42 and p-tau181/Aβ42 [38], which are included in the 2024 revised framework, unlike t-tau. Although t-tau was in the 2018 NIA-AA framework, its removal from the 2024 revised framework makes it difficult to assert that green tea may be protective against neurodegeneration, and the results for t-tau/Aβ42 and p-tau181/Aβ42 do not provide evidence for protection against the neuropathological hallmarks of AD.

In 2020, Samuelsson et al. [39] published a study on the relationship between adherence to four dietary patterns and several CSF biomarkers: Aβ42, p-tau, t-tau, and ratios of CSF biomarkers. The four dietary patterns were (1) Western diet, (2) Mediterranean diet, (3) high protein and alcohol, and (4) high total and saturated fat. Adherence to each dietary pattern was measured using diet history. The outcomes were measured as continuous and dichotomous variables. The sample consisted of cognitively normal adults aged 70 in Sweden (n = 269, 49% women, 37% APOE ε4 positive). Using linear regression, the researchers found no associations between adherence to any dietary pattern and any outcome. Using logistic regression (adjusted for sex, education, BMI, energy intake, and physical activity), the researchers found associations between higher adherence to the Western diet and odds of high t-tau (OR = 1.43, 95% CI = 1.02, 2.01, *p* = 0.04) but no association between any dietary pattern and Aβ42/Aβ40 [39], the only biomarker in the study that is also included in the 2024 revised framework.

In 2020, Tian et al. [40] published a study on the relationship between scores for “spicy food” consumption and several CSF biomarkers: Aβ42, p-tau, t-tau, and ratios of CSF biomarkers. “Spicy” refers to capsaicin and similarly hot spices. Spicy food consumption scores were measured using a four-question, SQ-FFQ asking about spicy foods only. The sample consisted of cognitively normal adults in China (n = 131; mean age, % female, and % APOE ε4 positive not reported). Using partial correlation (adjusted for age, sex, and education), the researchers found a positive association between higher spicy food consumption scores and Aβ42 (R = 0.325, *p* < 0.001) and negative associations between higher spicy food consumption and t-tau/Aβ42 (R = −0.181, *p* = 0.041) and p-tau181/Aβ42 (R = −0.223, *p* = 0.011). These main findings suggest that hot spices may be protective against early, soluble tau [40]. Notably, this was the only CSF study that found evidence of a relationship between diet and a neuropathological hallmark of AD, as defined by the 2024 revised framework.

In 2025, Chu et al. [41] published a study on the relationship between daily fresh fruit and vegetable consumption and several plasma and imaging biomarkers: Aβ42, Aβ40, Aβ42/40, t-tau, p-tau181, and NfL in plasma; and amyloid load and tau load, measured using PET. Daily fresh fruit and vegetable consumption was measured using a semi-quantitative questionnaire and converted to a binary outcome (high-moderate consumption vs. low consumption) for each subgroup of fresh fruits and vegetables. The ROIs for amyloid load were the frontal gyrus, lateral parietal gyrus, lateral temporal gyrus, medial temporal gyrus, posterior cingulate gyrus, and precuneus, whereas the ROIs for tau load included the entorhinal cortex, hippocampus, inferior temporal cortex, fusiform gyrus, and parietal and frontal lobes among others. The sample consisted of cognitively normal adults in China with positive results for amyloid load, i.e., presymptomatic but on the AD continuum (n = 177, mean age = 65 years, 56% female, 28% APOE ε4 positive). Notably, this is the only cross-sectional study in this review that used a biomarker of AD as an inclusion criterion. Using multiple linear regression (adjusted for age, sex, education, APOE ε4 status, cognitive scores, BMI, smoking, alcohol intake, and history of hypertension, diabetes mellitus, hyperlipidemia, and coronary heart disease), the researchers found negative associations for plasma p-tau181 with high-moderate dark vegetable consumption (β = −0.719, 95% CI = −1.249, −0.189, *p* = 0.008) and high-moderate grape consumption (β = −0.545, 95% CI = −0.928, −0.162, *p* = 0.006) among the participants who had plasma data (n = 138). For amyloid load (n = 177), the researchers found negative associations with high-moderate consumption of vegetables (β = −0.096, 95% CI = −0.171, −0.021, *p* = 0.012) and two fruit subgroups: berry (β = −0.162, 95% CI = −0.232, −0.093, *p* < 0.001) and grape (β = −0.132, 95% CI = −0.203, −0.061, *p* < 0.001). There were also negative associations with tau load (n = 53) for high-moderate consumption of fruit (β = −1.022, 95% CI = −1.776, −0.279, *p* = 0.009) and grapes (β = −0.856, 95% CI = −1.633, −0.078, *p* = 0.032). These main findings suggest fruits and vegetables may be protective against amyloid plaques and hyperphosphorylated tau, with grapes being protective against both hallmarks of AD [41].

In 2025, Mrhar et al. [42] published a study on the relationship between adherence to the Mediterranean diet and inflammation level of the diet and several plasma biomarkers: t-tau, p-tau181, Aβ42/Aβ40, NfL, and GFAP. Notably, NfL and GFAP are the only plasma biomarkers that are part of the 2024 revised framework. Adherence to the Mediterranean diet and inflammatory level of the diet were determined using a SQ-FFQ. The sample consisted of cognitively normal adults aged at least 60 years in Sweden (n = 1907, 60% female, 43% APOE ε4 positive). Using quantile regression (adjusted for age, sex, occupation, education, BMI, energy intake, physical activity level, smoking, and diagnosis with diabetes, hypertension, heart diseases, cerebrovascular disease, chronic lung disease, chronic kidney disease, anemia, cancer, depression and mood diseases), the researchers found a negative association between adherence to the Mediterranean diet and p-tau181 (β = −0.036, 95% CI = −0.072, −0.001, *p* < 0.05) and a positive association between inflammatory level of the diet and NfL (β = 0.031, 95% CI = 0.008, 0.053, *p* < 0.05) among those in at least the 75% percentile for plasma levels of p-tau181 and NfL. These main findings suggest that Mediterranean diet may protect against hyperphosphorylated tau in individuals who have high levels of plasma p-tau181 [42].

Overall, the cross-sectional studies suggest that several dietary exposures may be protective against the development of the neuropathological hallmarks of AD [34,35,37,40,41,42], while low protein intake is harmful, being associated with higher amyloid load [36]. However, a variety of dietary exposures were tested, with only three of the nine studies testing the same exposure- adherence to the Mediterranean diet [37,39,42]. These studies had different results, with one study suggesting that the Mediterranean diet may be protective against amyloid plaques [37], another study finding no association with any of the hallmarks of AD [39], and the most recent study finding no association for amyloid plaques but a negative association with hyperphosphorylated tau [42]. All three studies had different methods, with the first using a SQ-FFQ and imaging biomarkers [37], the second using the more detailed diet history method and CSF biomarkers [39], and the third using a SQ-FFQ and plasma biomarkers [42]. Methodological differences were also found across all nine cross-sectional studies, but all used a retrospective dietary measure, which is limited by recall bias. Two studies published in 2025 included plasma biomarkers [41,42], which were first introduced in the 2024 revised framework, but a 2018 study also included plasma biomarkers [36]. Although these studies tested plasma biomarkers for both plaques and tau, significant associations were only found for tau [41,42]. Among the CSF studies, the study by Tian et al. (2020) was the only one that found evidence of a relationship between diet and a neuropathological hallmark of AD, as defined by the 2024 revised framework [40]. The other two CSF studies [38,39] did include biomarkers from the 2024 revised framework, but they tested different exposures than Tian et al. (2020) [40]. Among the imaging studies, only one imaging study included a measure from group T [41], and all imaging studies had different ROIs for amyloid load [34,35,36,37,41] (Table 4). All nine cross-sectional studies differed in their covariates as well (Table 5), with only two studies controlling for physical activity [39,42] and five studies controlling for BMI [35,36,39,41,42]. Most studies did not examine physical activity or exercise at all [34,35,36,37,38,40,41]. Interestingly, only one study used a biomarker of AD as an inclusion criterion [41]. Although all studies were analytical cross-sectional studies, none mentioned a power analysis. In terms of funding sources, none were funded by food industry. Due to their design, all studies were limited by an inability to establish temporality and minimize residual confounding.

### 3.2. Cohort

Four studies had a cohort study design (n = 4), with Chu et al. (2025) [41] conducting both cross-sectional and longitudinal analyses (n = 1). All longitudinal analyses are presented in this sub-section in chronological order by year of publication and in alphabetical order by last name of the first author. A summary of the studies is presented in Table 6.

In 2018, Berti et al. [43] published a study on the relationship between adherence to the Mediterranean diet and three imaging biomarkers: (1) amyloid load, measured using PET, (2) glucose metabolism, measured using FDG-PET, and (3) gray matter volume, measured using MRI. Adherence to the Mediterranean diet was measured using a SQ-FFQ. The ROIs were not defined in the methods section, but the regions that showed significant results were presented in the results section. The sample consisted of cognitively normal adults in NYC (n = 70, mean age = 50 years, 67% female, 39% APOE ε4 positive). After a mean follow-up (f/u) of 3 years (with adjustment for age, sex, education, APOE ε4 status, and time to f/u), lower adherence to the Mediterranean diet was associated with greater increases in amyloid load in the frontal cortex and posterior cingulate cortex/precuneus regions (pinteraction < 0.001) and greater decreases in glucose metabolism in the temporal and posterior cingulate cortex/precuneus regions (pinteraction < 0.002) compared to higher Mediterranean diet adherence. This suggests that the low adherence to the Mediterranean diet may increase the risk of amyloid plaques and neurodegeneration [43].

In 2018, Rainey-Smith et al. [44] published a study also investigating the relationship between adherence to the Mediterranean diet and amyloid load, measured using PET. Adherence to the Mediterranean diet was determined from a SQ-FFQ. The ROIs were the frontal, superior parietal, lateral temporal, occipital and anterior and posterior cingulate regions. The sample consisted of cognitively normal adults in Australia with a relatively high amyloid load at baseline or a high rate of beta-amyloid accumulation during f/u (n = 77, mean age = 71 years, 49% female, 42% APOE ε4 positive). After a f/u of 3 years (with adjustment for age, sex, APOE ε4 status), higher adherence to the Mediterranean diet was associated with a decrease in amyloid load (β = −0.01, *p* = 0.007), suggesting a protective effect of the Mediterranean diet against future amyloid plaque accumulation [44].

In addition to the cross-sectional results by Chu et al. (2025) [41] reported in this review, the same study included a longitudinal analysis. After a f/u of almost 2.5 years (with adjustment for age, sex, education, APOE ε4 status, cognitive scores, BMI, smoking, alcohol intake, and history of hypertension, diabetes mellitus, hyperlipidemia, and coronary heart disease), there were negative associations with amyloid load for high-moderate consumption of vegetables (β = −0.287, 95% CI = −0.412, −0.162, *p* < 0.001) and berries (β = −0.162, 95% CI = −0.308, −0.016, *p* < 0.032) for the subset of the sample that had amyloid load data (n = 24). For the subset that had plasma data (n = 14), no significant associations were found after a f/u of almost 2 years. Notably, the sample sizes in the longitudinal analysis were much smaller (less than 25) than those in the cross-sectional analysis, likely reducing power to detect differences [41].

In 2025, Hoost et al. [45] published a study on the relationship between fatty acid intake and several plasma biomarkers: p-tau181, Aβ42/Aβ40, p-tau181/Aβ42, GFAP, and NfL. Fatty acid intake was measured using a SQ-FFQ and converted into tertiles of intake for MUFAs, omega-3 fatty acids, and omega-6 fatty acids. The sample consisted of cognitively normal adults in New York City (n = 599, mean age = 74 years, 70% female, 25% APOE ε4 positive). After a mean f/u of 7 years (with adjustment for age, sex, education, ethnicity, storage time, energy intake, and cardiovascular risk), there were negative associations with p-tau181 for those in the highest tertile for omega-3 fatty acid intake and the highest tertile of omega-6 fatty acid intake compared to those in the lowest tertiles (β = −0.030, *p* = 0.027; β = −0.047, *p* = 0.002; respectively). Additionally, there was a negative association between omega-6 fatty acid intake and GFAP, an indicator of neurodegeneration, when comparing those in the highest tertile of omega-6 fatty acid intake to those in the lowest tertile (β = −0.028, *p* = 0.009). These main findings suggest that both omega-3 fatty acids and omega-6 fatty acids may be protective against hyperphosphorylated tau [45].

In 2025, Rajendra et al. [46] published a study on the relationship between dietary nitrate intake and two imaging biomarkers: (1) rate of amyloid load deposition, measured using PET, and (2) rate of gray matter volume atrophy, measured using MRI. Dietary intake was measured using a SQ-FFQ and converted into tertiles of dietary nitrate intake. The ROIs were not specified. The sample had a median age of 71 and was stratified by sex and APOE ε4 status, with some of the sample having PET scans (n = 554), most of whom also had MRI scans (n = 335). After a maximum f/u of 10.5 years (with adjustment for age, time, time by dietary nitrate, marital status, education, BMI, energy intake, physical activity, and smoking status), there were negative associations with rate of amyloid load deposition for female APOE ε4 carriers in the highest tertile of plant-sourced dietary nitrate intake compared to those in the lowest tertile. However, for male APOE ε4 carriers and female APOE ε4 non-carriers, a significant negative association was only observed when comparing the middle tertile to the lowest. These main findings provide support for precision nutrition interventions for AD prevention, with dietary nitrate intake showing promise as a potential protective factor in the highest risk group by sex and APOE ε4 status [46].

Overall, the cohort studies, like the cross-sectional studies, tested different exposures, with only two studies testing the same exposure: the Mediterranean diet [43,44]. But unlike the cross-sectional studies, these studies both found a protective effect of the Mediterranean diet against amyloid load. They both measured amyloid load using PET and used a SQ-FFQ to determine adherence to the Mediterranean diet. Furthermore, the f/u in both studies was 3 years, and similar sample sizes were used but drawn from different populations (NYC and Australia). Only one of those studies reported their power analysis [43], and it was the only cohort study to do so. To measure the exposure variable, all the cohort studies used a SQ-FFQ, which is limited by recall bias. Three studies included imaging biomarkers only [43,44,46], one included plasma biomarkers only [45], and one included both imaging and plasma biomarkers [41]. None included CSF biomarkers. Furthermore, none of the imaging studies measured tau load using PET. Like in the cross-sectional studies, the imaging studies differed in their selection of ROIs to measure amyloid load [41,43,44], and each study used different covariates, with only two studies controlling for BMI [41,46] and one controlling for physical activity [46]. Most did not examine physical activity or exercise at all [41,43,44,45]. Half of the cohort studies used an AD biomarker as an inclusion criterion [41,44], and only one study measured change in cognitive status as one of the outcomes [41]. Like the cross-sectional studies, none of the studies were funded by food industry. Unlike the cross-sectional studies, these studies establish temporality, but they are still limited by residual confounding, necessitating randomized trials.

### 3.3. Intervention

Only one intervention study met the inclusion/exclusion criteria for this review: In 2021, Hoscheidt et al. [47] published a double-blind, randomized trial on the effects of the Mediterranean and Western diets on several CSF biomarkers, among other outcomes, including cognitive outcomes. The CSF biomarkers were Aβ40, Aβ42/Aβ40, t-tau, and Aβ42/t-tau. The dietary interventions were isocaloric but differed in their saturated fat, glycemic index, and sodium levels, with the levels being higher in the Western diet. Compliance to the intervention was measured using diet records and described as follows: “[c]ompliance was excellent with an average of <1 non-compliant meal per week.” The sample consisted of two groups: cognitively normal adults and adults with mild cognitive impairment (MCI). The results for adults with MCI will not be presented here. The cognitively normal adults were in North Carolina and Washington (n = 56, mean age = 50s, 71% female, APOE ε4 not reported). After 4 weeks of the intervention (adjusting for age, sex, APOE ε4 status, BMI, cognitive scores, and site), there was a significant difference in the mean change in Aβ42/Aβ40 between the Mediterranean diet and Western diet intervention groups (*p* = 0.014): while Aβ42/Aβ40 increased on the Mediterranean diet, it decreased on the Western diet. There was no significant difference in the effect of the diets on t-tau/Aβ42, the only other biomarker in this study that is also included in the 2024 revised framework. These main findings suggest that the Mediterranean diet may be an effective intervention against amyloid plaque formation, while the Western diet may increase amyloid plaque formation [47]. However, the study is limited by its small sample size, missing power analysis results, short duration of the intervention, and lack of control for physical activity. Like all intervention studies, its limitations also include limited external validity and generalizability.

## 4. Discussion

The reviewed literature suggests that a variety of dietary factors may prevent the formation of the neuropathological hallmarks of AD in cognitively normal adults, suggesting that diet may prevent AD or the progression of AD from a pre-symptomatic phase to a symptomatic phase. However, each potentially protective or harmful dietary factor needs to be tested in more studies. The most tested factor was the Mediterranean diet, tested in three cross-sectional studies [37,39,42], two cohort studies [43,44], and one intervention study [47]. Two out of the three cross-sectional studies suggest a protective effect of the Mediterranean diet [37,42], also reported in both cohort studies [43,44] and the intervention study [47]. These findings are like those reported by systematic reviews and meta-analyses showing a relationship between diet and prevention of AD [5,6,7,8,9,30,48,49,50], including a relationship between the Mediterranean diet and prevention of AD [31,51,52]. Interestingly, none of the studies included in our review tested the MIND diet, although it is specifically designed to reduce the risk of neurogenerative diseases like AD and has been found to be protective against dementia in a 2025 meta-analysis [10]. Future studies should consider investigating the relationship between the MIND diet and the development of the neuropathological hallmarks of AD. Also, future studies should consider studying the many traditional diets of the Global South, as the current evidence base is dominated by studies on the Global North and on a version of the Mediterranean diet that reflects Eurocentric perspectives [53]. For example, all the studies on the Mediterranean diet in our review were on populations in the Global North [37,39,42,43,44,47].

There are several studies suggesting a link between the Mediterranean diet and AD, though most of the research is observational with AD incidence or cognitive performance as the primary outcome, and the mechanisms are not yet fully elucidated. Among study participants without clinical AD, a 2025 meta-analysis of observational studies found a negative association between adherence to the Mediterranean diet and AD incidence [52], and a sub-study of the PREDIMED-NAVARRA randomized trial found improvements in cognitive performance when comparing 6.5 years on a Mediterranean diet with additional olive oil or a Mediterranean diet with additional nuts compared to a diet low in fat [54]. Among participants with clinical AD, a 2025 cohort study found reduced decline in cognitive function with adherence to the Mediterranean diet, though it is not expected to treat AD but instead manage it [55]. Several components and traits of the Mediterranean have been hypothesized to explain the link with AD prevention. These mechanisms include reduced neuroinflammation due to polyphenols, omega-3 fatty acids, and effects of fiber on the microbiome [56] and reduced oxidative stress and amyloid and tau load due to polyphenols [57], including oleocanthal in extra virgin olive oil [58]. Furthermore, the Mediterranean diet has positive effects on cardiovascular disease and insulin resistance [59], major risk factors of AD. In fact, a 2024 report from the *Lancet* Commission lists hypertension, high LDL cholesterol, and diabetes as three of fourteen risk factors responsible for many of the AD cases worldwide [60], and some researchers have started to refer to AD as type III diabetes, with the AD brain showing insulin resistance [61].

The cross-sectional studies in this review tested various exposures and employed various methods. The only exposure tested in more than one cross-sectional study was the Mediterranean diet, and there was disagreement in the results [37,39,42]. This may be due to differences in the methods, including variability in assessment of adherence to the Mediterranean diet (SQ-FFQ or diet history) and assessment of biomarkers, with each study choosing a different measurement modality (imaging, CSF, or plasma). This heterogeneity in methods makes it difficult to draw conclusions on the relationship between the Mediterranean diet, or any diet tested in more than one study, and the development of the neuropathological hallmarks of AD in cognitively normal adults. All in all, more studies testing the same exposure are needed, but also comparable methods and outcome measures are needed to facilitate assessment of consistency, a criterion in the Bradford-Hill criteria for causation that is necessary for generalization of study findings.

The cohort studies in this review also tested various exposures and differed in their methods, including their f/u times. However, comparable methods and the same f/u time were used to evaluate the Mediterranean diet, the only exposure tested in more than one cohort study [43,44]. There was agreement in the findings, increasing confidence in the generalizability of the protective effect of the Mediterranean diet against amyloid plaques. While the f/u times were the same for the two cohort studies on the Mediterranean diet, f/u times ranged widely from 2.5 years [41] to 10.5 years [46]. Notably, the pre-symptomatic stage of AD is estimated to range from 15–20 years [62]. F/u times shorter than 15 years make it difficult to distinguish delay of symptoms vs. prevention of AD. All in all, more cohort studies are needed to test the same specific exposure over longer f/u times.

Surprisingly, many of the studies in this review did not control for physical activity and BMI, despite the evidence linking physical activity and BMI to both diet and AD [63], and none of the studies controlled for estrogen levels or menopausal status, which may be linked to AD [64]. Among the nine cross-sectional analyses, only two studies controlled for physical activity [39,42], and five studies controlled for BMI [35,36,39,41,42]. Among the five cohort analyses, one controlled for physical activity [46], and one controlled for BMI [41]; none controlled for both. In the intervention study, there is no information on the physical activity level of the participants, but the study does compare participants in the different groups on BMI, finding no significant differences. This inconsistency in control of physical activity and BMI is especially surprising given the 2020 report from the *Lancet* Commission, which lists physical inactivity and obesity as modifiable risk factors of dementia [65]. Also, two randomized trials have shown protective effects of multidomain lifestyle interventions, which include both diet and physical activity [11,12]. As a result, not controlling for physical activity jeopardizes the internal validity of any study on diet and AD prevention. Proper selection of covariates is critical for increasing internal validity.

In addition to more studies testing the same exposures and using the NIA-AA biomarker-based definition of AD, future research should prioritize prospective cohort and randomized trial designs, as most of the current evidence base consists of analytical cross-sectional studies. Also, future research could consider including a combination of CSF, imaging, and plasma outcome measures if feasible as well as more measures of hyperphosphorylated tau. Additionally, future research would benefit from consensus on ROIs, controlled variables, and inclusion/exclusion criteria to facilitate comparison of findings. Using biomarkers as inclusion/exclusion criteria would also help discern primary prevention strategies from secondary prevention strategies. Furthermore, future studies should include power analyses and consider methods of dietary assessment other than retrospective methods, including weighed food records and biomarkers of dietary intake. All in all, the NIA-AA framework represents an exciting opportunity to progress our knowledge on prevention of AD, and the current research suggests a potential role for diet in AD prevention, but more research is needed to address the current gaps in the evidence base and develop evidence-based recommendations for primary and secondary prevention of AD.

This systematic narrative review has several strengths. First, to the best of our knowledge, this is the first review on diet and AD prevention that is focused on cognitively normal adults and AD-specific biomarkers from the 2018 NIA-AA framework and 2024 revised framework. Second, it employed a systematic search strategy, describing the methods used to search for the literature included in the review, allowing for replication studies. Third, it identifies current limitations in the evidence base and provides recommendations for future research.

However, this systematic narrative review also has some limitations. First, a limited number of databases were included in the review, although PubMed (MEDLINE) is one of them and considered the most relevant database for medical research. Second, the review did not involve grading the quality of the evidence. Third, the review did not weigh the strength of the evidence. Finally, as in all systematic narrative reviews, there is a potential for author bias.

## 5. Conclusions

The recent NIA-AA framework defined AD using biomarkers, facilitating research into primary and secondary prevention strategies. Reviewing the literature that has used biomarkers from this framework, the evidence suggests existence of a potential role for dietary interventions, especially the Mediterranean diet, in AD prevention among cognitively normal adults. However, the evidence base is small with heterogeneity in the methods. Consensus on methods and further research are needed to address existing gaps and develop dietary recommendations for primary and secondary prevention of AD.

## Figures and Tables

**Table 1:** 2018 NIA-AA Framework.

Group	Biomarkers
A	Low Aβ42 or Aβ42/Aβ40 in CSF Cortical amyloid in PET
T	High hyperphosphorylated tau (p-tau) in CSF Cortical tau in PET
(N)	High total tau (t-tau) in CSF Hypometabolism in FDG-PET Atrophy in MRI

**Table 2:** 2024 Revised Framework.

Group	Biomarkers
A	Low Aβ42/Aβ40 in CSF Cortical amyloid in PET
T_1_	High p-tau181/Aβ42, t-tau/Aβ42 in CSF High p-tau217 in plasma
T_2_	High MTBR-tau243, other p-tau, mid-region tau fragments * in CSF Cortical tau in PET High MTBR-tau243, other p-tau in plasma
(N)	High neurofilament light chain (NfL) in CSF or plasma Hypometabolism in FDG-PET Atrophy in MRI
(I)	High glial fibrillary acidic protein (GFAP) in CSF or plasma
(V)	Infarction White matter hyperintensity
(S)	Synuclein seeding

* Non-phosphorylated.

**Table 3:** Summary of Cross-Sectional Studies (n = 9).

Author (Year)	Sample	Exposure (Instrument)	Outcomes (Modality)	Limitations
Mosconi et al. (2014) [34]	NYC n = 49 mean age = 54 69% female 39% APOE ε4 +	SFAs, MUFAs, ω-3, ω-6, β-carotene, B12, C, D, E, folate (SQ-FFQ)	↑ B12, ↑ EPA → ↓ amyloid (imaging)	Temporality, residual confounding, recall bias, sample size, generalizability
Berti et al. (2015) [35]	NYC n = 52 mean age = 54 71% female 47% APOE ε4 +	B + minerals, MUFAs + PUFAs + E, A + C + carotenoids + DF, B12 + D + Zn, SFA + trans-fat + chol + Na (SQ-FFQ)	↑ B12 + D + Zn → ↓ amyloid (imaging)	Temporality, residual confounding, recall bias, sample size, generalizability
Fernando et al. (2018) [36]	Australia n = 541 mean age = 70 59% female 26% APOE ε4 +	Protein, DF (SQ-FFQ)	↓ protein → ↑ amyloid (imaging) No significant findings for AD hallmarks (plasma)	Temporality, residual confounding, recall bias, generalizability
Vassilaki et al. (2018) [37]	Minnesota n = 278 mean age = 78 44% female 27% APOE ε4 +	Mediterranean diet (SQ-FFQ)	↑ Mediterranean diet → ↓ amyloid (imaging)	Temporality, residual confounding, recall bias, generalizability
Ma et al. (2020) [38]	China n = 722 mean age = 62 40% female 15% APOE ε4 +	Green tea (single question)	No significant findings for AD hallmarks (CSF)	Temporality, residual confounding, exposure assessed as frequency only, recall bias, generalizability
Samuelsson et al. (2020) [39]	Sweden n = 269 age = 70 49% female 37% APOE ε4 +	Western diet, Mediterranean diet, High protein + alcohol, High total fat + SFA (diet history)	No significant findings for AD hallmarks (CSF)	Temporality, residual confounding, recall bias, generalizability
Tian et al. (2020) [40]	China n = 131 mean age = ? ? % female ? % APOE ε4 +	Spicy foods (SQ-FFQ)	↑ spicy food → ↑ Aβ42, ↓ t-tau/Aβ42, ↓ p-tau181/Aβ42 (CSF)	Temporality, residual confounding, sample size, recall bias, generalizability, Aβ42 is not in 2024 framework
Chu et al. (2025) [41]	China n = 177 mean age = 65 56% female 28% APOE ε4 +	Daily fresh fruit, vegetables (SQ-questionnaire)	↑ dark vegetables, ↑ grapes → ↓ p-tau181 (plasma) ↑ vegetables, ↑ berries, ↑ grapes → ↓ amyloid (imaging) ↑ fruit, ↑ grapes → ↓ tau (imaging)	Temporality, residual confounding, sample size, recall bias, generalizability
Mrhar et al. (2025) [42]	Sweden n = 1907 age = 60+ 60% female 43% APOE ε4 +	Mediterranean diet, inflammatory level of the diet (SQ-FFQ)	↑ Mediterranean diet → ↓ p-tau181 (plasma)	Temporality, residual confounding, sample size, recall bias, generalizability

**Table 4:** ROIs for Amyloid Load in Imaging Cross-Sectional Studies (n = 5).

Author (Year)	ROIs
Mosconi et al. (2014) [34]	Inferior parietal lobe, lateral temporal lobe, medial frontal gyrus, posterior cingulate cortex, prefrontal cortex
Berti et al. (2015) [35]	Inferior parietal, superior parietal, lateral temporal, medial temporal, posterior cingulate cortex, prefrontal cortex, striatum
Fernando et al. (2018) [36]	Frontal, superior parietal, lateral temporal, and lateral occipital lobes; anterior and posterior cingulate cortex
Vassilaki et al. (2018) [37]	Parietal and temporal lobes, prefrontal and orbitofrontal cortices, posterior cingulate cortex, precuneus
Chu et al. (2025) [41]	Frontal gyrus, lateral parietal gyrus, lateral temporal gyrus, medial temporal gyrus, posterior cingulate gyrus, precuneus

**Table 5:** Controlled Variables in Cross-Sectional Studies (n = 9).

Author (Year)	Controlled Variables
Mosconi et al. (2014) [34]	Age, energy intake
Berti et al. (2015) [35]	Sex, education, APOE ε4, ethnicity, BMI, family history, alcohol intake
Fernando et al. (2018) [36]	Age, sex, education, APOE ε4, Australia vs. another country, BMI, energy intake
Vassilaki et al. (2018) [37]	Age, sex, education, APOE ε4, time from FFQ to PET, energy intake
Ma et al. (2020) [38]	Age, sex, education, APOE ε4, cognitive scores
Samuelsson et al. (2020) [39]	Sex, education, BMI, energy intake, physical activity
Tian et al. (2020) [40]	Age, sex, education
Chu et al. (2025) [41]	Age, sex, education, APOE ε4 status, cognitive scores, BMI, smoking, alcohol intake, and history of hypertension, diabetes mellitus, hyperlipidemia, and coronary heart disease
Mrhar et al. (2025) [42]	Age, sex, occupation, education, BMI, energy intake, physical activity level, smoking, and diagnosis with diabetes, hypertension, heart diseases, cerebrovascular disease, chronic lung disease, chronic kidney disease, anemia, cancer, depression and mood diseases

**Table 6:** Summary of Cohort Studies (n = 5).

Author (Year)	Sample	Exposure (Instrument)	Outcomes (Modality)	Limitations
Berti et al. (2018) [43]	NYC n = 70 mean age = 50 67% female 39% APOE ε4 + f/u = 3 years	Mediterranean diet (SQ-FFQ)	↓ Mediterranean diet → ↑ amyloid (imaging)	Residual confounding, recall bias, sample size, duration of f/u, generalizability
Rainey-Smith et al. (2018) [44]	Australia n = 77 mean age = 71 49% female 42% APOE ε4 + f/u = 3 years	Mediterranean diet (SQ-FFQ)	↑ Mediterranean diet → ↓ amyloid (imaging)	Residual confounding, recall bias, sample size, duration of f/u, generalizability
Chu et al. (2025) [41]	China n = 24 mean age = ? ? % female ? % APOE ε4 + f/u = 2.5 years	Daily fresh fruit, vegetables (SQ-questionnaire)	↑ vegetables, ↑ berries → ↓ amyloid (imaging) No significant findings for AD hallmarks (plasma)	Residual confounding, recall bias, sample size, duration of f/u, generalizability
Hoost et al. (2025) [45]	NYC n = 599 mean age = 74 70% female 25% APOE ε4 + f/u = 7 years	MUFAs, ω-3, ω-6 (SQ-FFQ)	Highest tertile for ω-3, ω-6 → ↓ p-tau181 (plasma)	Residual confounding, recall bias, duration of f/u, generalizability
Rajendra et al. (2025) [46]	Australia n = 554 median age = 71 f/u = 10.5 years	Plant nitrate intake (SQ-FFQ)	Highest tertile for nitrate → ↓ amyloid among female APOE ε4 carriers (imaging)	Residual confounding, recall bias, loss of power due to stratification, duration of f/u, generalizability

## Data Availability

No new data were created or analyzed in this study. Data sharing is not applicable to this article.

## Abbreviations

The following abbreviations are used in this manuscript:

AD	Alzheimer’s Disease
APOE	Apolipoprotein E
ε4	Epsilon 4
ACh	Acetylcholine
AChE	Acetylcholinesterase
APP	Amyloid Precursor Protein
ROS	Reactive Oxygen Species
NIA-AA	National Institute on Aging and Alzheimer’s Association
CSF	Cerebrospinal Fluid
PET	Positron Emission Tomography
MRI	Magnetic Resonance Imaging
NfL	Neurofilament Light Chain
GFAP	Glial Fibrillary Acidic Protein
DASH	Dietary Approaches to Stop Hypertension
MIND	Mediterranean-DASH Intervention for Neurodegenerative Delay
SQ-FFQ	Semi-Quantitative Food Frequency Questionnaire
SFA	Saturated Fatty Acid
MUFA	Monounsaturated Fatty Acid
EPA	Eicosapentaenoic Acid
ROI	Region of Interest
PUFA	Polyunsaturated Fatty Acid
DF	Dietary Fiber
BMI	Body Mass Index
F/U	Follow-Up
MCI	Mild Cognitive Impairment
